# “Electronic Phenotyping” Antimicrobials to Facilitate Outpatient Stewardship for Asymptomatic Bacteriuria and Urinary Tract Infection in Renal Transplant

**DOI:** 10.1093/ofid/ofae119

**Published:** 2024-03-14

**Authors:** Alex N Zimmet, David Ha, Emily Mui, Mary Smith, Marten Hawkins, William Alegria, Marisa Holubar

**Affiliations:** Division of Infectious Diseases and Geographic Medicine, Stanford University School of Medicine, Stanford, CA, USA; Stanford Antimicrobial Safety and Sustainability Program, Stanford Health Care, Stanford, CA, USA; Division of Infectious Diseases and Geographic Medicine, Stanford University School of Medicine, Stanford, CA, USA; Stanford Antimicrobial Safety and Sustainability Program, Stanford Health Care, Stanford, CA, USA; Division of Infectious Diseases and Geographic Medicine, Stanford University School of Medicine, Stanford, CA, USA; Stanford Antimicrobial Safety and Sustainability Program, Stanford Health Care, Stanford, CA, USA; Division of Infectious Diseases and Geographic Medicine, Stanford University School of Medicine, Stanford, CA, USA; Stanford Antimicrobial Safety and Sustainability Program, Stanford Health Care, Stanford, CA, USA; Division of Infectious Diseases and Geographic Medicine, Stanford University School of Medicine, Stanford, CA, USA; Stanford Antimicrobial Safety and Sustainability Program, Stanford Health Care, Stanford, CA, USA; Division of Infectious Diseases and Geographic Medicine, Stanford University School of Medicine, Stanford, CA, USA; Stanford Antimicrobial Safety and Sustainability Program, Stanford Health Care, Stanford, CA, USA; Division of Infectious Diseases and Geographic Medicine, Stanford University School of Medicine, Stanford, CA, USA; Stanford Antimicrobial Safety and Sustainability Program, Stanford Health Care, Stanford, CA, USA

**Keywords:** antibiotic prescribing, billing data, immunocompromised, renal transplant, urinary tract infection

## Abstract

Asymptomatic bacteriuria and urinary tract infection in renal transplant are important antimicrobial stewardship targets but are difficult to identify within electronic medical records. We validated an “electronic phenotype” of antibacterials prescribed for these indications. This may be more useful than billing data in assessing antibiotic indication in this outpatient setting.

Implementation of antimicrobial stewardship principles is increasingly necessary within solid organ transplant (SOT) given rising rates of antimicrobial resistance and infection with multidrug-resistant organisms [1, 2]. However, few data exist to guide evaluation of stewardship initiatives in outpatient SOT clinics. Asymptomatic bacteriuria (ASB) and urinary tract infection (UTI) in renal transplant (RT) represents one domain in which antimicrobial stewardship programs (ASPs) may seek to optimize outpatient antibiotic use in SOT given high-quality data to guide stewardly practices [2, 3].

*International Classification of Diseases, Tenth Edition* (ICD-10), data are commonly used by ASPs to infer the indication for antibiotic prescriptions in primary care settings [4–8]. This has not been validated in SOT clinics, where billing practices are not well described, antibiotic prophylaxis is common, and many clinical problems are addressed concurrently. Additionally, many centers (including ours) do not require documenting an indication in the electronic order when prescribing outpatient antimicrobials. In this study we aimed to (1) characterize ICD-10 use in RT clinic encounters in which antibacterials were prescribed, (2) develop a new metric termed “acute urinary antibiotics” (AUAs) using “electronic phenotyping” [9, 10] of prescriptions to track treatment of ASB and UTI in this population, and (3) validate AUAs to enable practical implementation.

## METHODS

We retrospectively reviewed all encounters in the RT clinic at Stanford Health Care (including telephone and telemedicine visits) from January 2018 to December 2021, extracting dates, ICD-10 data, and antimicrobial prescription data. We analyzed encounters within 6 months of RT in which antibacterials were prescribed because this is the highest risk period for UTI and the most likely time frame in which ASB may be treated [11, 12]. We divided ICD-10 data into 4 categories: “Post-Transplant Care”; “Urinary Infection/Colonization” (which included ASB); “Other Acute Infection/Antibiotic Indication”; and “Non-Infectious/Miscellaneous.”

We categorized antibacterials as AUAs if they met the following 3 criteria: (1) dosed at least every 48 hours for a total duration of ≤28 days (those not meeting these criteria were deemed prophylaxis); (2) drug or drug class routinely prescribed for UTI (Supplementary Table 1); and (3) had no associated ICD-10 for a nonurinary infection. We expressed the AUA prescribing rate as days of therapy per 1000 patient days, summing all recipients’ days of follow up within the study period (capped at 6 months after RT) as the denominator. For validation, 1 author (A.Z.) performed chart review on all prescriptions from 2021 to assess clinician-documented antibacterial indication and, in cases with confirmed intent to treat a urinary syndrome, presence of urinary symptoms. We calculated the positive percent agreement (PPA) of AUA as the percent of prescriptions with documented intent to treat ASB or UTI classified as AUAs, and negative percent agreement (NPA) as the percent of prescriptions with documented alternative intent classified as non-AUAs [13]. We also calculated PPA and NPA for Urinary Infection/Colonization ICD-10 data for comparison. For both AUA and ICD-10, we calculated 95% confidence intervals (CI) and the overall kappa statistic compared with documented antibacterial indication (R statistical software, version 4.3.2., R Foundation for Statistical Learning, Austria).

## RESULTS

**Figure 1.**
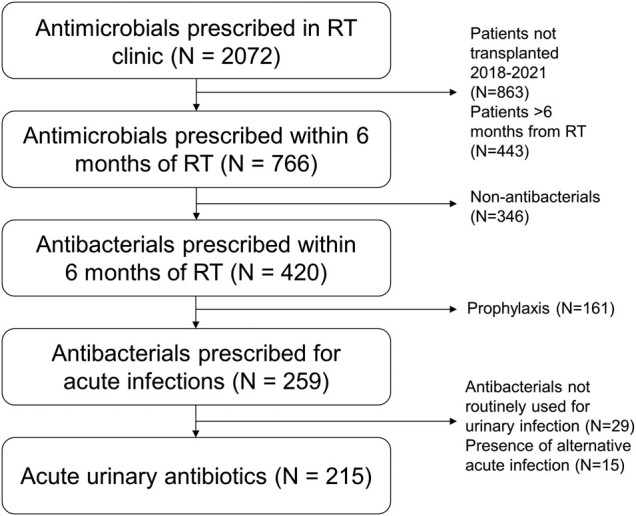
Classification of outpatient antibiotic prescriptions for patients who received renal transplant, 2018–2021. RT, renal transplant.

Our center performed 608 RTs from 2018 through 2021. We reviewed 2072 antimicrobial prescriptions (Figure 1) ordered at 1895 encounters (451/1895 [24%] office visit or telemedicine encounters; 1444/1895 [76%] extra-visit encounters [eg, telephone calls]). After limiting to 6 months after RT, our analytic cohort included 420 antibacterials (402 individual encounters, 135/402 [33%] office visit or telemedicine) among 238 unique patients (136 [57%] male, 112 [47%] Hispanic/Latino).

A total of 624 ICD-10s were used across these 402 encounters (mean 1.6 per encounter); 244 encounters had multiple ICD-10s. Posttransplant care comprised 74% (461/624) of all ICD-10s (Supplementary Table 2). ICD-10s for urinary infection/colonization (94/624 [15%]) and other acute infections (31/624 [5%]) were used sparingly.

We classified 215/420 (51%) prescriptions as AUAs, the majority being fluoroquinolones (80/215, 37%) and cephalosporins (60/215, 28%) (Supplementary Figure 1). The AUA prescribing rate within 6 months of RT was 19.8 AUA days of therapy per 1000 patient-days.

The 2021 validation cohort included 130 antibacterials—59/130 (45%) were classified as AUAs and 51/130 (39%) had documented intent to treat ASB or UTI. Only 15/51 (29%) had urinary symptoms; 32/51 (63%) were asymptomatic and 4/51 did not describe whether symptoms were present. AUA agreed with documented intent in 49/51 cases of ASB/UTI treatment (PPA 96%; 95% CI, 91–100) and 69/79 cases of alternative indications (NPA 87%; 95% CI, 80–95) with high overall agreement (kappa = 0.82; Table 1). ICD-10 had a higher NPA (77/79 [97%]; 95% CI, 95–100) but lower PPA (21/51 [41%]; 95% CI, 28–55) and overall agreement (kappa = 0.53; Table 2).

**Table 1.** Concordance of “Acute Urinary Antibiotics” Classification With Clinician-Documented Intent to Treat ASB or UTI

		Intent to Treat ASB/UTI	
		Yes	No
Acute urinary antibiotics	Yes	49	10
	No	2	69

Abbreviations: ASB, asymptomatic bacteriuria; AUA, acute urinary antibiotics; RT, renal transplant; UTI, urinary tract infection.

**Table 2.** Concordance of ICD-10 With Clinician-Documented Intent to Treat ASB or UTI

		Intent to Treat ASB/UTI	
		Yes	No
Urinary infection/colonization ICD-10	Yes	21	2
	No	30	77

Abbreviations: ASB, asymptomatic bacteriuria; AUA, acute urinary antibiotics; ICD-10, *International Classifications of Diseases, Tenth Edition;* RT, renal transplant; UTI, urinary tract infection.

## DISCUSSION

We found infrequent use of UTI or ASB ICD-10 data in RT encounters with antibacterial prescriptions. We developed and validated AUA, an alternative metric that accurately identified antibacterial prescriptions intended to treat UTI or ASB in RT patients. This suggests novel metrics can assist antimicrobial tracking by indication in SOT and other settings where use of ICD-10 for this purpose is limited.

We found little heterogeneity amongst ICD-10 data associated with outpatient antibacterial prescriptions in this cohort, with the majority centered on routine posttransplant care. Despite this, there were clearly various antibacterial indications not reflected in the ICD-10 data because we classified 51% of all antibacterials prescribed as AUAs. Outpatient ASPs commonly use ICD-10 to identify antimicrobial indications in primary care settings (eg, by reviewing encounters coded for “viral upper respiratory infection” or “urinary tract infection”). In transplant clinics, however, multiple concurrent indications for antibiotics (including prophylaxis) may exist. We found that billing data did not adequately reflect this complexity, although management of these patients is complex, coding appears oversimplified. This suggests ICD-10 data idiosyncrasies may preclude their utility in measuring indication-specific antibiotic prescribing in this setting.

AUA reliably differentiated antibacterials intended for opportunistic infection prophylaxis and those prescribed for ASB or UTI in RT based on “electronic phenotyping” [9, 10] (eg, algorithmically interpreting data inherent to the prescription to determine its indication). This exhibited better overall agreement with chart-documented indication compared with ICD-10 alone, suggesting that ASPs working with transplant clinics may benefit from this novel approach to tracking indication-specific antimicrobial use.

ASB was commonly treated in our cohort despite emerging data to support withholding antibiotics for this condition. AUAs, many of which were fluoroquinolones, made up a majority of antibacterials given in the RT clinic, and 63% of AUAs in the validation cohort were given to asymptomatic patients. Several randomized controlled trials [14–17] have shown no benefit (and possible harm) from this practice, which is now discouraged by multiple societies [18, 19]. Thus, AUA classification may help ASPs dependably identify a large pool of syndrome-specific, broad-spectrum antibacterial use in the outpatient RT setting. This could then be examined to identify patterns of excess prescribing (eg, routine treatment of ASB), guide creation of evidence-based local guidelines, and track subsequent practice changes. As ASPs expand to ambulatory and immunocompromised domains, such data can help foster new relationships with transplant teams and initiate collaborative efforts to optimize antimicrobial use in these high-risk patients.

Our study has limitations. First, we did not evaluate the appropriateness of individual prescriptions. Indeed, AUA does not distinguish between treatment of ASB versus UTI, and therefore the “goal” AUA prescribing rate is unclear (though likely not zero). However, many ASP metrics (eg, days of antimicrobial therapy) have similar limitations, yet serve as benchmarks of antimicrobial prescribing. Second, we did not adjudicate whether each prescription represented initial empiric therapy or a switch to targeted therapy, which could overestimate the true AUA prescribing rate; nevertheless, we felt this was most representative of how an ASP would pragmatically implement this metric. Conversely, by focusing on outpatient prescriptions, AUAs begun during a hospitalization may not have been captured, potentially underestimating the true AUA prescribing rate. Fourth, we did not perform sensitivity analyses to optimize specific AUA criteria (eg, testing AUA when defined as ≤30 days’ duration instead of ≤28 days); such validation could further enhance the precision of this metric. Fifth, this was a single-center study at an academic medical center; our findings would benefit from validation in other settings. Finally, we limited our study to those within 6 months of RT; applicability to patients further out from transplant should be explored.

## CONCLUSIONS

ICD-10 data may not accurately measure outpatient antibacterial prescribing for ASB and UTI in RT patients. We developed and validated AUA, an alternative metric that appropriately identified antibiotic prescriptions intended to treat UTI or ASB in RT patients and which ASPs may find more useful than billing data for this purpose. Electronic phenotyping such as AUA represents a pragmatic alternative means of measuring indication-specific antimicrobial use. Innovative strategies for measuring antimicrobial prescribing data are needed to further develop antimicrobial stewardship in immunocompromised hosts.

## Supplementary Material

ofae119_Supplementary_Data

## Supplementary Data

Supplementary materials are available at *Open Forum Infectious Diseases* online. Consisting of data provided by the authors to benefit the reader, the posted materials are not copyedited and are the sole responsibility of the authors, so questions or comments should be addressed to the corresponding author.

## Notes

***Acknowledgments***. We thank Patrick Ahearn, Colin Lenihan, and Jane Tan for their collaboration and partnership.

***Patient consent statement.*** This quality improvement project was deemed nonhuman subjects research by the Stanford Panel on Human Subjects in Medical Research.

***Financial support***. Research reported in this publication was supported by the National Center For Advancing Translational Sciences of the National Institutes of Health under Award Number UL1TR003142.
