# Supplementary material for: “Electronic Phenotyping” Antimicrobials to Facilitate Outpatient Stewardship for Asymptomatic Bacteriuria and Urinary Tract Infection in Renal Transplant
Source: Open Forum Infect Dis. 2024 Mar 14;11(3):ofae119. doi: 10.1093/ofid/ofae119 (PMC10964979; doi:10.1093/ofid/ofae119)
Supplement: ofae119_Supplementary_Data [file ofae119_supplementary_data.zip › Supplemental Material.docx]

Supplemental Figure 1. Proportion of Acute Urinary Antibiotic Use in Renal Transplant Clinic within 6 Months of Transplant by Antibacterial Class, 2018-2021. AUA, Acute urinary antibiotics.

Supplemental Table 1. Drugs/Drug Classes Defined by Routine Use for Urinary Tract Infections.

| **Drug/Drug Class** | **Routinely Used for UTI?** |
| --- | --- |
| Penicillins | Yes |
| Cephalosporins | Yes |
| Fluoroquinolones | Yes |
| Trimethoprim-sulfamethoxazole | Yes^#^ |
| Fosfomycin | Yes |
| Tetracyclines (doxycycline) | Yes |
| Macrolides (azithromycin) | No |
| Isoniazid | No |
| Antivirals | No |
| Antifungals | No |
| Clindamycin | No |
| Dapsone | No |
| Metronidazole | No |
| Fidaxomicin | No |
| Hydroxychloroquine | No |
| Vancomycin | No |

^#^149/153 prescriptions for trimethoprim-sulfamethoxazole met criteria for prophylaxis and thus were not eligible to be considered AUAs.

Abbreviations: UTI, urinary tract infection.

Supplemental Table 2. Categorization of ICD-10s Used in RT Clinic Encounters in which Antibacterials Were Prescribed

| **Disease Condition** | **ICD-10** | **Number of encounters** |
| --- | --- | --- |
| **Post-transplant care ICD-10s** |  | **461** |
| Kidney transplant status | Z94.0 | 321 |
| Encounter for aftercare following kidney transplant | Z48.22 | 55 |
| Immunosuppression | D84.9 | 37 |
| Immunosuppressive management encounter following kidney transplant | Z79.899 | 31 |
| Aftercare following organ transplant | Z48.298 | 5 |
| Pancreas replaced by transplant | Z94.83 | 4 |
| Need for prophylactic antibiotic | Z79.2 | 4 |
| Transplant recipient | Z94.89 | 3 |
| Complication of transplanted kidney, unspecified complication | T86.10 | 1 |
| **Urinary infection/colonization** |  | **94** |
| Urinary tract infection without hematuria, site unspecified | R82.813 | 41 |
| Abnormal finding on urinalysis | R82.90 | 11 |
| Acute cystitis without hematuria | N30.00 | 10 |
| Dysuria | R30.0 | 10 |
| Pyuria | R82.81 | 6 |
| Urinary tract infection with hematuria, site unspecified | R82.84 | 5 |
| Acute cystitis with hematuria | N30.01 | 2 |
| Pyelonephritis of transplanted kidney | T86.19 | 2 |
| Need for prophylaxis against urinary tract infection | Z29.8 | 2 |
| Acute pyelonephritis | N10 | 1 |
| Acute UTI | N39.0 | 1 |
| Bacteriuria | R82.71 | 1 |
| Urine culture positive | R82.79 | 1 |
| At risk of UTI | Z91.89 | 1 |
| **Other Acute Infection/Antibiotic Indication** |  | **31** |
| *Clostridioides difficile* colitis | A04.72 | 8 |
| Cellulitis, unspecified cellulitis site | L03.90 | 6 |
| Cellulitis of left finger | L03.012 | 5 |
| Infection of arteriovenous fistula, subsequent encounter | T82.7XXD | 3 |
| Fever, unspecified cause | R50.9 | 2 |
| Ear infection | H66.90 | 1 |
| Pneumonia due to infectious organism, unspecified laterality, unspecified part of lung | J18.9 | 1 |
| Dental infection | K04.7 | 1 |
| Abscess of right arm | L02.413 | 1 |
| Finger wound, simple, open, initial encounter | S61.209A | 1 |
| Wound infection after surgery | T81.49XA | 1 |
| Multiple drug resistant organism culture positive | Z16.24 | 1 |
| **Non-infectious/miscellaneous** |  | **38** |
| Essential hypertension | I10 | 6 |
| Type 2 diabetes mellitus with stage 5 chronic kidney disease, unspecified long term insulin use | E11.22 | 4 |
| Type 2 diabetes mellitus with diabetic nephropathy, unspecified long term insulin use | E11.21 | 3 |
| Hyperkalemia | E87.5 | 2 |
| Renovascular hypertension | I15.0 | 2 |
| Arthralgia, unspecified joint | M52.50 | 2 |
| Other neutropenia | D70.8 | 1 |
| Post-transplant diabetes mellitus | E13.9 | 1 |
| Secondary hyperparathyroidism | E21.1 | 1 |
| Hypophosphatemia | E83.39 | 1 |
| Hypercalcemia | E83.52 | 1 |
| Calciphylaxis | E83.59 | 1 |
| Bilateral dry eyes | H04.123 | 1 |
| Heart failure with reduced ejection fraction | I50.2 | 1 |
| ESRD | N18.6 | 1 |
| Asplenia | Q89.01 | 1 |
| RUQ pain | R10.11 | 1 |
| Nausea | R11.0 | 1 |
| Diarrhea, unspecified type | R19.7 | 1 |
| Memory impairment | R41.3 | 1 |
| Seizures | R56.9 | 1 |
| Impaired glucose tolerance | R73.02 | 1 |
| Acute kidney injury | S37.0 | 1 |
| Need for prophylactic vaccination against viral disease | Z23 | 1 |
| History of splenectomy | z90.81 | 1 |

Abbreviations: ICD-10, *International Classification of Diseases, Tenth Revision*; RT, renal transplant; UTI, urinary tract infection; ESRD, end-stage renal disease; RUQ, right upper quadrant.
