# Supplementary figures and images for: “Electronic Phenotyping” Antimicrobials to Facilitate Outpatient Stewardship for Asymptomatic Bacteriuria and Urinary Tract Infection in Renal Transplant
Source: Open Forum Infect Dis. 2024 Mar 14;11(3):ofae119. doi: 10.1093/ofid/ofae119 (PMC10964979; doi:10.1093/ofid/ofae119)

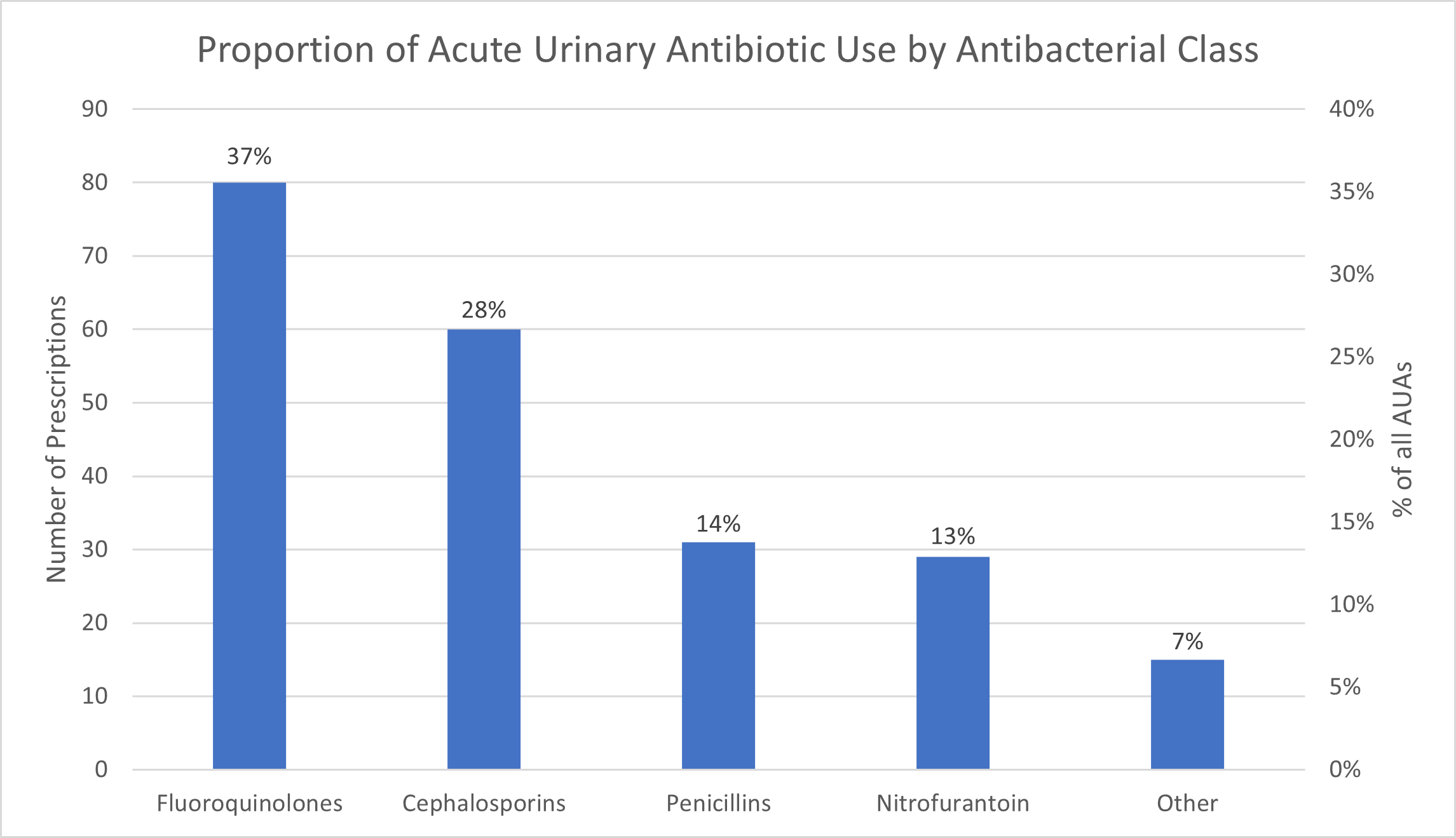

Supplement: ofae119_Supplementary_Data [file ofae119_supplementary_data.zip › Supp Fig 1.tif]
